# Rosai-Dorfman Disease: Self-Resolving Unilateral Lymphadenopathy and a Brief Review of Literature

**DOI:** 10.1155/2018/4869680

**Published:** 2018-09-16

**Authors:** Joshua Feriante, Richard T. Lee

**Affiliations:** ^1^Lincoln Memorial University-DeBusk College of Osteopathic Medicine, 6965 Cumberland Gap Pkwy, Harrogate, TN 37752, USA; ^2^Tennessee Cancer Specialists, 1415 Old Weisgarber Rd #200, Knoxville, TN 37909, USA

## Abstract

Rosai-Dorfman disease (RDD), also known as sinus histiocytosis with massive lymphadenopathy (SHML), is a disease of histiocytic proliferation with no known pathogenesis. This disease is defined by histological and cytological characteristics, including emperipolesis and positive S100 and CD68 markers. Although the disease typically presents clinically with massive bilateral lymphadenopathy due to sinus expansion from excessive histiocytosis, only unilateral lymphadenopathy was observed in this patient. The case involves a 40-year-old Caucasian male from the Netherlands presenting with unilateral lymphadenopathy suspicious for malignancy. Subsequent histological and laboratory testing led to the rare diagnosis of RDD. The lymphadenopathy resolved spontaneously over the course of several weeks following the initial presentation. Rosai-Dorfman disease reportedly has a benign prognosis with approximately 20% of patients experiencing spontaneous disease resolution (as was the case for this patient) with 70% experiencing chronic symptoms that may last years but not require intervention. We therefore advocate observation as a mainstay of treatment for most cases of this rare disease with intervention only being pursued in symptomatic cases. A review of recent literature regarding pathogenesis, epidemiology, diagnostic factors, prognosis, and treatment is provided and discussed.

## 1. Introduction

Rosai-Dorfman disease (RDD), also known as sinus histiocytosis with massive lymphadenopathy (SHML), is a disease of benign histiocytic proliferation of unknown etiology [1, 2]. Many hypotheses have been proposed to explain the cause of RDD or SHML with the two leading causes being autoimmune dysregulation or histiocytic proliferation secondary to infection. The disease is currently diagnosed based on histological and cytological characteristics, the hallmark diagnostic factors being emperipolesis (discussed below) and positive S100 and CD68 markers [1–6]. Negative CD1a markers have been useful in differentiating them from other diseases [1–4, 6]. Despite how little is known of the disease, the prognosis is generally favorable with half of diagnosed cases resolving spontaneously resulting in no or few adverse sequelae [1, 3, 5–7]. Of cases that do warrant treatment, steroids and surgery have been shown to be effective in treating more severe sequelae of the disease, including obstruction or compression of vital organs, symptomatic lymphadenopathy, and fever [1, 2, 4–8].

Much is still unknown about RDD or SHML, yet the current understanding provides a precursory framework that may one day lead to a more standardized model of care for diagnosed patients. A review of the current literature is included to discuss salient topics regarding pathogenesis, diagnosis, treatment, and prognosis of this rare disease.

## 2. Case Presentation

A 40-year-old male originally from the Netherlands presented to the clinic with an acute onset of unilateral left cervical adenopathy that began approximately three months prior. The lymphadenopathy was uncomfortable but not reported as painful. Prior medical history includes several kidney stones, and the patient as a child underwent tonsillectomy and tympanostomy tubes. The patient had never smoked, and a review of the family history did not reveal any instances of cancer. No other signs or symptoms were present to suggest lymphoma or leukemia, and the patient denied fever, chills, night sweats, or weight loss. The patient denied headache, seizures, or any other neurological deficits that would have indicated extra nodal central nervous system involvement [9]. Spontaneous reduction of lymphadenopathy was observed on a subsequent visit three weeks later. A long-term follow-up was discussed and arranged with the patient to follow the course of the disease.

CT imaging demonstrated a 3 cm lymph node in the left level II region as well as several 1 cm left jugulodigastric nodes. Additionally, a PET scan of the torso (from skull base to thigh) was performed after the injection of 17.4 mCi of fluorine-labeled fludeoxyglucose and revealed the left level II and jugulodigastric nodes with a maximum standardized uptake value of 3.0. No other significant metabolic activities or lymphatic involvement was seen in the chest, abdomen, or pelvis.

**Figure 1:**
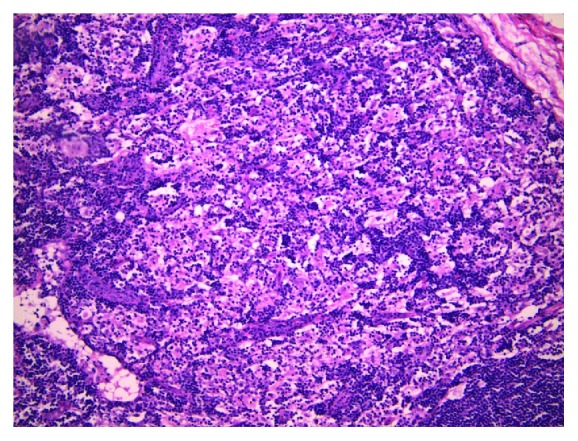
Section with hematoxylin and eosin stain under 100x magnification showing excessive hyperplasia of large histiocytes with abundant clear to pink cytoplasm and intracytoplasmic lymphocytes.

**Figure 2:**
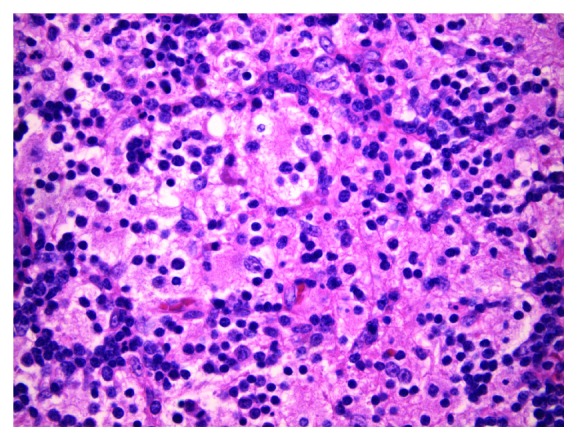
Section with hematoxylin and eosin stain under 400x magnification. Emperipolesis is the phagocytosis of cells such as lymphocytes and erythrocytes within another cell such as a histiocyte. This is a characteristic of Rosai-Dorfman disease and was evident on histopathology of the tissue biopsy.

The patient was referred for excisional biopsy, and the tissue was reviewed by a team of hematopathologists. The excised lymph nodes measured 3.3 × 2 × 1.3 cm and 1 × 0.7 × 0.2 cm. Histological section revealed native architecture distortion by excessive numbers of large histiocytes with abundant foamy cytoplasm (Figure 1). No histiocytic atypia was observed, and acid-fast staining and GMS fungal staining were negative. Reed-Sternberg cells and well-defined granulomas were absent. The nodes were stained with a S100 protein monoclonal 4C4.9 antibody at a dilution of 1 : 50 which revealed numerous S100-positive histiocytes. The nodes were free of signs of malignancy. Emperipolesis, one of the few definitive diagnostic criteria for the disease, was evident on microscopy (Figure 2).

Laboratory results of the collected complete blood count, complete metabolic panel, and lactate dehydrogenase labs were all within normal limits. Serum protein electrophoresis detected IgG kappa monoclonal proteins of low concentration.

## 3. Discussion

Due to the rarity of RDD or SHML, no definitive pathogenesis has been identified. There have been multiple proposed hypotheses, with infectious and autoimmune disorders being the most prevalent [1, 4, 6, 10]. Both human herpes virus-6 (HHV-6) and parvovirus B19 have been suspected as the leading causes of histiocytic proliferation and have been associated with several case studies [1, 4, 6, 10]. Interestingly, HHV-6 antigens have been detected in histiocytes in some cases, whereas parvovirus B19 and Epstein-Barr virus (EBV) antigens and protein capsids have been positive in lymphocytes which may then later be phagocytosed by histiocytes [6, 10]. This suggests that the histiocytic proliferative response is secondary to B or T cellular infection by parvovirus B19 or another immunological trigger [5, 10]. Alternatively, several studies have not detected an association of parvovirus B19 or HHV-6 with RDD [6]. Moreover, histiocytes have been shown to be HHV-6 positive in the absence of disease, which has led some authors to preclude herpes virus or parvovirus from being the definite cause of RDD or SHML [6]. Other infectious agents that have been associated with RDD include Epstein-Barr virus, cytomegalovirus, Brucella, and Klebsiella [1, 4]. The exact infectious mechanism is unknown, yet there has been speculation that histiocytic proliferation may be mediated by a macrophage colony-stimulating factor [2, 4].

Speculation of autoimmune dysregulation as the possible cause of RDD or SHML has little substantial evidence. Mehraein et al. proposed that RDD or SHML may be an acquired autoimmune disorder in which cellular apoptotic signaling pathways are disrupted leading to a histiocytic proliferation [10]. RDD was thought to be associated with IgG4-related diseases due to several studies of RDD with extranodal involvement in the pulmonary system. However, there is little evidence that they share common pathological causes [6].

Several genetic mutations have been recently identified in histiocyte disorders, including NRAS, KRAS, and MAP2K1 mutations. KRAS and MAP2K1 are associated with 33% of RDD cases, which suggests that the RAS-MAP2K1 pathway may be an important component of disease development and may be used for targeted therapy [11].

According to recent studies, cutaneous Rosai-Dorfman disease (CRDD) has been proposed as a distinct disease from RDD due to several pathological differences [6, 12]. Such differences include exclusive involvement of the skin with possible nodal involvement in CRDD [12]. Demographic features differ considerably with the average age of CRDD diagnosis at 45 years and a predisposition among females, whereas the average age of the diagnosis of RDD is 20 years with a male predominance [4, 6–8]. Moreover, most cases of CRRD have been reported in Caucasian and Asian populations. RDD has an increased incidence among African and West Indian populations [6–8]. Finally, CRDD is associated with a better prognosis compared to RDD [12]. The absence of coalescing pigmented papulonodular clusters, verrucous plaques, nodules, or satellite lesions ruled out a diagnosis of cutaneous Rosai-Dorfman disease (CRDD) in this case [13].

The clinical presentation of RDD has several predictable signs and symptoms, the most salient being painless bilateral lymphadenopathy reported in nearly 90% of cases with the cervical region being the most commonly involved nodal site in 90% of RDD patients with lymphadenopathy [1, 3, 5, 7, 10]. Several cases have been reported in which lymphadenopathy was unilateral or minimal [1, 7, 8, 14]. Additionally, fever is reported in 30% of RDD patients [1]. Although RDD can present in any age group and is considered a worldwide disease, it most typically presents in childhood or young adulthood with an estimated average age of onset at 20 years [2–4, 6–8, 10].

Extranodal involvement is observed in approximately 40% of cases and can occur anywhere in the body with the most common sites including the skin, gastrointestinal tract, eyes, external and internal ear, skeletal system, upper and lower respiratory tracts, oral cavity, nasal and paranasal cavities, and the central nervous system [6–8]. Central nervous system (CNS) involvement is often the most difficult to treat and often requires surgery to relieve compression of brain structures as RDD commonly manifests as dural-based masses [9]. A recent review of cases of RDD with CNS involvement showed that 79.5% of cases had intracranial lesions, 11.4% had spinal involvement, and 9.0% presented with both intracranial and spinal involvements [9]. Symptoms are largely dependent on the location of the extranodal involvement with the most common symptoms mimicking other tumors of the CNS including headache, seizures, focal neurologic deficits, and focal paralysis if the spine is involved [9].

There have been several cases of extranodal involvement of the mediastinum, liver, and spleen, although such cases are exceedingly rare [2, 15].

Several laboratory findings are useful in diagnosing RDD. Specifically, the erythrocyte sedimentation rate (ESR) is elevated in nearly 90% of RDD cases and polyclonal hypergammaglobulinemia is present in approximately 80–90% of cases according to several studies [1, 4–8]. Other less common laboratory findings associated with RDD include elevated C-reactive protein (CRP), anemia, neutrophilia, leukocytosis, thrombocytopenia, arthropathy, arthritis, and elevated rheumatoid factor [1, 4, 5, 7, 8, 10, 14, 16]. The types of anemias are many and may include normocytic or microcytic varieties [2]. Autoimmune hemolytic anemia is an uncommon reported finding [4–6]. Eosinophilia has also been reported but is a rare finding [6].

There are several immunohistochemical markers that are integral to establishing a diagnosis of RDD or SHML with the most important markers being S100 and CD68. Other utilized markers include HAM56, CD14, CD64, CD15, lysozyme, alpha-1 antitrypsin, and IL-2 [1–6]. CD163 has recently been recognized as a marker of histiocytes that is positive in SHML and may prove useful in diagnosing RDD [2, 5, 6]. RDD consistently produces negative results for Langerhans cell marker CD1a and dendritic cell markers DRC, CD23, and CNA42 [1–4, 6].

Histology is essential in establishing a diagnosis of RDD or SHML. Lymph nodes are described as possessing dilated sinuses surrounded by capsular and pericapsular fibrosis [3–5]. The nodal architecture is preserved in some cases while it is distorted due to extensive sinus dilation in others [5, 6]. The most common finding on microscopy is an excess of large histiocytes with abundant pale eosinophilic cytoplasm which may occasionally take on a foamy appearance. Emperipolesis, also known as lymphophagocytosis, is the hallmark diagnostic finding of RDD or SHML and is defined as the phagocytosis of cells (including lymphocytes and erythrocytes) that remain undamaged and able to move within another cell such as a histiocyte [5, 6]. These histological features are typically present on a background of lymphocytes, plasma cells, neutrophils, and a scarce number of eosinophils [6, 8].

The prognosis of SMHL or RDD is generally benign with a relatively large number of reported spontaneous regressions. The eventual outcome is often unpredictable, however. Recent studies suggest that up to 20% of cases of RDD or SMHL regress spontaneously without therapy while approximately 70% of patients experience chronic disease that often manifests in a remitting and relapsing pattern that can potentially last for years [1–3, 5–7, 9]. Moreover, spontaneous resolution appears to favor cases with nodal involvement only [5]. Mortality from diagnosed RDD or SHML is associated with an extra nodal development of disease in vital organs such as the CNS and kidneys or from complications associated with immunosuppression or amyloidosis [2, 16].

There is currently no standard treatment protocol for RDD or SHML due to its rarity and lack of clinical trial data [6, 9]. Observation is generally the mainstay of treatment due to spontaneous regression without complication in approximately half of patients who are diagnosed with RDD or SHML [1–3, 5–8]. However, medical treatment is often necessary when extranodal involvement obstructs or compresses vital organs, particularly to maintain airway patency or to prevent compression of cerebral or spinal structures [1, 4, 6–9]. In cases of symptomatic lymphadenopathy, fevers, and swelling, steroids are effective in reducing symptoms and have been proposed as first-line therapy [3, 5, 6, 8]. A wide variety of treatments have been attempted, including chemotherapy (including methotrexate, 6-mercaptopurine, cladribine, and isotretinoin), cryotherapy, topical chemotherapy, and topical isotretinoin. Although all such treatments have generally been proven ineffective [3, 5–7, 11], a recent case of perirenal RRD associated with a KRAS mutation was treated with cobimetinib with substantial improvement [11]. Radiotherapy has been proposed as an option for palliative treatment to control symptoms in cases where surgery may not be feasible [6].

## 4. Conclusion

Rosai-Dorfman disease, or sinus histiocytosis with massive lymphadenopathy, is a rare disease of histiocytic proliferation of unknown etiology that often mimics other diseases and malignancies and can easily be misdiagnosed. It is diagnosed by observing emperipolesis on histology, positive S100 and CD68 markers, and negative CD1a marker. Because the disease generally resolves spontaneously, observation is currently the advocated approach to treating RDD or SHML. Although chemotherapy, cryotherapy, and isotretinoin have been used unsuccessfully, steroids or surgery has been effective in treating complications associated with extra nodal disease.

## Abbreviations

RDD: Rosai-Dorfman disease
SHML: Sinus histiocytosis with massive lymphadenopathy
CRDD: Cutaneous Rosaid-Dorfman disease
CNS: Central nervous system
CT: Computed tomography
CRP: C-reactive protein
PET: Positron emission tomography
HHV-6: Human herpes virus-6
EBV: Epstein-Barr virus
ESR: Erythrocyte sedimentation rate.
